# "Not From the College, but Through the Public and the Legislature": Charles Maclean and the Relocation of Medical Debate in the Early Nineteenth Century

**DOI:** 10.1353/bhm.0.0078

**Published:** 2008

**Authors:** Catherine Kelly

**Keywords:** Charles Maclean, plague, contagion, select committees

## Abstract

Charles Maclean is generally thought to have played an important role in the contagion debates of the early nineteenth century and to have prompted two parliamentary inquiries into the issue. The author examines the effects of Maclean's efforts to relocate the contagion debates from the medical to the public sphere. The author shows that Maclean's tactics challenged the exclusivity of medical knowledge by ceding power to decide the debate to a non-medically expert Parliament. The author also demonstrates how this conflict laid bare the side-by-side existence of two probative systems in medical debates during the early nineteenth century by examining the medical profession's struggle to establish what type of evidence would be considered probative and what type of witness would be considered competent to give it.

This gentleman has been described by an enlightened member of parliament, as one of those extraordinary persons who will be pointed out by the finger of the future historian! History has two fingers . . . which of those two she will use, if she ever happen to notice Dr M'Lean, we will not venture to predict.^1^

Charles Maclean, the gentleman referred to in the preceding quotation, has featured prominently in the history of anticontagionism.^2^ Historians[Other P-545] believe that his contribution to the contagion debates of the early nineteenth century, particularly his investigation of the plague in Constantinople, ^3^ caused Parliament to establish a Select Committee in 1819 to investigate "the validity of the Doctrine of Contagion in the Plague" and a further inquiry in 1824 into the effect of quarantine on Britain's foreign trade.^4^

Most historians who have written on the subject of contagion debates in the nineteenth century have focused on the period after 1825. Their works principally concentrate on the political motivations behind state responses to the threat of epidemic disease and on the tensions between quarantine and free trade. Events and actors from the early 1800s—particularly Maclean—are used to support the argument that the contagion debates were not only a struggle between scientific or medical theories but were caused by, or were representative of, social, economic, and political movements and conflicts. They treat the anticontagionist movement of the early nineteenth century and the parliamentary debates of the 1820s[Other P-546] as important background but, after identifying there the seeds and character of later anticontagionism, most historians do not engage in further discussion of the early 1800s.

Three of the most important works on the contagion debates, Erwin Ackerknecht's seminal essay, "Anticontagionism Between 1821 and 1867,"^5^ Margaret Pelling's *Cholera, Fever and English Medicine 1825-1865*,^6^ and Roger Cooter's "Anticontagionism and History's Medical Record"^7^ stress the importance of social and political ideologies to the medical debate on contagion, and each gives some consideration to the early nineteenth century.^8^ Despite the works' significant differences, to varying extents they each identify early anticontagionists with political radicalism and present a liberal versus conservative analysis of the British contagion debates, particularly in the years before 1830.^9^ These historians all identify Maclean as an important leader of the anticontagionist movement and instigator of the parliamentary inquiries.

Each of them also cites Maclean as evidence of the connection between radical politics and anticontagionism. Ackerknecht uses Maclean to support his argument that the anticontagionists were led by radicals and liberals.^10^ Pelling portrays Maclean as the leader of an anticontagionist movement opposed by the establishment. She describes Maclean as "anti-authoritarian" and closely allied with liberals, radicals, and free-marketers opposed to quarantine.^11^ Cooter attributes radical political views to anti-contagionists throughout the century, again using Maclean, "the person mainly responsible for the introduction of anticontagionism into Britain," to support this argument.^12^ Cooter also uses this early period to argue[Other P-547] for the radical underpinnings of the movement: "in the first third of the nineteenth century anticontagionists were attempting first and foremost through anticontagionism to challenge the social and cultural impositions of the old order."^13^ Other authors have written about events after 1825 but have said very little about Maclean, his politics, or the early debates.^14^

In this paper, I challenge the characterization of Maclean as a physician inspired to anticontagionism by his radical politics and opposition to quarantine. I argue, rather, that Maclean's anticontagionism developed early in his medical career, independent of his radical politics, and that he incorporated his politics (anti-authoritarianism, an opposition to quarantine, and hostility to the Catholic Church) into his anticontagionist position only after his efforts to achieve prominence in the medical world and make anticontagionism the prevailing orthodoxy were unsuccessful. There is significant evidence to suggest that this incorporation was a deliberate act by Maclean, intended to relocate the debates on contagion from the medical sphere to the public sphere, where he believed he would have a better chance of achieving his goals. Maclean's success in locating the debate in the public sphere represented a direct threat to a large group of "elite" practitioners who asserted the medical profession's exclusive right to consider and determine medical questions and bore directly on what types of evidence and argument were considered probative in medical debates.

## The Development of Maclean's Anticontagionism

Historians who have considered the influence of Maclean have drawn one-dimensional representations of him as a radical, agitator, or mercantilist. Although most argue that Maclean was responsible for the introduction of anticontagionism in Britain, his theories regarding epidemic diseases are rarely mentioned.

Maclean did not often refer to "anticontagionism" but instead to his theory of "epidemic and pestilential disease," of which anticontagionism formed an important part. For the first two decades of his practice, Maclean's medical theories were relatively independent of his politics, and it is during this period that he developed his theory of epidemic and pestilential disease. Accordingly, to understand the nature of Maclean's[Other P-548] anticontagionism it is necessary to examine separately the development of his medical and political philosophies and then consider how he incorporated the latter into the former. This will demonstrate that the issue of quarantine was not central to the formation of Maclean's anticontagionism.

Maclean's medical career began in India in the late 1780s. After serving aboard various East Indiamen, he practiced in Calcutta for several years and during this time was also the publisher of a newspaper.^15^

While many of Maclean's medical works focused on the issue of contagion, he devoted significant time and energy to other medical causes. The range of his medical interests is demonstrated in his early medical works, in which (among other things) he strongly opposed the practice of bleeding,^16^ advocated the extensive use of mercury,^17^ and involved himself with a controversial antivaccination group.^18^ In each case, he set out his opinions forcefully and attacked medical traditionalists; however (perhaps despite his self-image), it would not be accurate to describe Maclean's medical theories as idiosyncratic or even especially radical.

Maclean was "greatly influenced"^19^ by John Brown's *Elementa Medicinae,* in which Brown identified a single cause of disease, attributing all states of health to changes in "excitability."^20^ Brunonianism dictated conclusions that Maclean made the bedrock of his medical system, in particular the belief in the singularity of fevers, the use of stimulants, and an opposition to specifics. Maclean complained only that Brown did not go far enough. In his earliest medical work (written in the 1790s) he took it upon himself to "correct" Brown's errors, the main issue being a denial of Brown's disease classification, "excessive excitement," which Maclean instead identified as an indirect form of debility.^21^

Maclean's medical practice was also influenced by his working environment in India. Although the description of Maclean as a rebel against the "medical establishment" is in many senses accurate, it implicitly fails to acknowledge the large number of medical practitioners who could be similarly characterized—including many of those men who made up the medical department of the East India Company. Within this tradition, the characteristic aspects of Maclean's medical theory, especially the use of mercury, the importance of atmosphere to disease, and skepticism regarding contagion, were not especially remarkable.^22^ During his time in Calcutta, Maclean worked closely with a Dr. William Yates,^23^ and together they wrote *A View of the Science of Life*.^24^

The departure of overseas practitioners, including Maclean, from traditional medical practices is explained in part by the challenging circumstances they encountered in tropical climates and also by the relative freedom from social and medical conventions offered so far from home. An empirical, ontological approach to medicine was facilitated by the availability and acceptability of postmortem investigation.^25^ Maclean's early professional medical experience was nurtured in this environment, and he cherished the flourishing culture of empiricism and experiment. In later life, he referred back to this period, praising the "free enquiry" encouraged "in the East India Company's extensive medical establishment."^26^

The third important influence on Maclean's early medical career was a strong philosophical commitment to the importance of truth and objectivity in science and to an empirical method of reasoning. He argued that his own work was premised on the doctrine that, just like all other parts of the universe, the human body was subject to "regular and immutable laws" that had previously, anomalously, not been applied to it. He believed that the "age of reason [had] begun to enlighten the medical world" and put much hope in the discovery of constant laws governing living beings.^27^ Maclean placed great stock in the obligation of the scientist or doctor to shoulder the "responsibility of innovation," arguing that not to do so was selfish and cowardly.^28^

These three factors directed Maclean's early medical work. His commitment to them was even recognized by his detractors in later years, despite their conviction that his zeal was "wrong directed" [*sic*].^29^ These were the principles that nurtured his anticontagionism, not an opposition to quarantine.

It is important to recognize the comprehensive nature of Maclean's theory on epidemic and pestilential disease in his early work. After acknowledging his debt to Benjamin Rush's account of the yellow fever in Philadelphia, he began his treatise on disease by setting out his definition of "contagion": "a specific matter, generated in a person affected with disease, and capable of communicating that particular disease, with or without contact, to another."^30^ He argued that plague, dysentery, and fevers, despite having been considered contagious for some time, were not; only a very few diseases such as smallpox and measles were contagious. ^31^ From these diseases, he identified the characteristics of contagious disease: it could affect people only once in their lifetimes^32^ and would always manifest in exactly the same way.^33^ After setting out his reasons for finding against contagion in most cases, he then identified the real cause of epidemic disease: the vicissitudes of the atmosphere in combination with other factors affecting the excitement of a person.^34^ He supported his argument by advancing the negative consequences of a belief in contagion resulting from the abandonment of the sick: higher mortality and the destruction of society's moral and familial ties.^35^ Even in this supporting section of his work, Maclean made only passing reference to the "folly" of quarantine.^36^ The issue of quarantine, if mentioned at all,[Other P-551] received similarly cursory treatment in his other writings, both medical and political, prior to 1816.^37^

Maclean did not rise to the top of the medical profession on the strength of his early publications. After the publication of *A View of the Science of Life*, his politics and newspaper writings brought him into conflict with the Marquis of Wellesley, resulting in Maclean's deportation from India. After his return to England, he traveled to the continent, where in 1802, the French government rejected his request for a grant to study contagion. His proposal, though deemed interesting, was rejected as being "of more interest to countries who have commercial connections with the Levant."^38^ At that time, Maclean conceded the importance of trade but, idealistically, argued that the advancement of science was a worthy end in itself, putting it ahead of commercial concerns.^39^

Maclean was held under Napoleon's detention of the English in France, and after his eventual release, he returned to England, where he joined the hospital staff of the British Army in the lowly position of hospital mate. This post, and the discrimination he experienced as a medical practitioner not trained at Oxford or Cambridge, was a disappointment to Maclean. His attempts to resign were refused by the Army Medical Board, and his departure from the service resulted (to his outrage) in his name being gazetted as a deserter in the *Hue and Cry*.^40^ Not surprisingly, Maclean soon afterward established himself as one of the Army Medical Board's most vocal critics.^41^

Until this point, Maclean's efforts to advance his career had been unsuccessful. His efforts appear to have turned next to the East India Company. In 1798, Maclean had published a pamphlet regarding the East[Other P-552] India Company (or, at least, the company under Wellesley) in which he had identified and condemned its monopolistic intentions.^42^ In contrast, in 1813, he published two works expressing admiration for the Company and argued for the protection of its monopoly.^43^ Despite qualifying this argument to support only a monopoly on navigation to India, it is evident that Maclean's political position had "evolved." In November 1810, the Company had given support to a series of lectures given by Maclean in London on the diseases of hot climates.^44^

From that time on it appears that Maclean cultivated the patronage of the Duke of Kent, the Levant Company, Lord Grenville, and the East India Company.^45^ His subsequent research in the Levant and his medical publications gave prominence to the idea of the "evils" of quarantine. This shift in emphasis may reflect a maturation of ideas; however, it is also perhaps indicative of the emergence of a willingness to adapt his position to appeal to his target audience and benefactors.

This conclusion is further supported by Maclean's association with the *Medical Observer*, a journal whose editors sought to expose "frauds and quacks." The *Medical Observer* treated Maclean more favorably than any other periodical. It advertised his lectures^46^ and printed his very long letters.^47^ The editors of the journal remained anonymous, but a public address by the disgruntled "original editor" in September 1808 indicated strongly that Maclean was, for a time, a joint editor of the journal. The accusations made by the "original editor" about the "new editor" (Maclean) were serious. He alleged that "[the new editor] was a disappointed and violent man" who insisted that "communications, respecting a certain *City* Practitioner, were [to be] omitted, that no offence might be given to him, because [the new editor] entertained expectations from him in the East India Company!!"^48^ If true, these allegations suggest that[Other P-553] Maclean was not by this time (1808) averse to giving his personal ambitions priority over other considerations.

This is not evidence of a complete abandonment of principles by Maclean. Instead, his association with the Company, the memory of his experience in France, and his continuing ostracism from the British medical elite together seem to have resulted in a fusion of his political and medical positions. In 1810, Maclean himself gave the clearest statement of his frustrations and new approach to the promotion of all his medical theories: "it can scarcely be necessary to say that it is not from the College, but through the public and the legislature, that I expect any favourable change to be effected in the profession of medicine."^49^

How, then, did Maclean attempt to court the "public and the legislature" regarding anticontagion? By giving prominence to the issues of quarantine and to the involvement of the Catholic Church in propagating the doctrine of contagion, and by telling an exciting story in which the hero, Maclean, contracts and survives the plague. In addition, by virtue of the legislative battlefield it occupied, "quarantine" provided an opportunity to score a tangible hit against the medical establishment for which he had formed a significant enmity.^50^

By focusing on trade and Catholicism, Maclean was drawing on two of the most prominent contemporary issues on the British agenda. During the later stages of the Napoleonic Wars, and after her victory, Britain's quickly growing economy was unstable and accompanied by frequent depressions. In addition, Britain's national debt continued to rise, totaling £792 million by 1816.^51^ Consequently, debates on appropriate economic policy occupied the public mind.^52^ The Catholic question, perennially an evocative one, continued to vex the Liverpool government and rouse strong public emotions throughout this period.^53^

These themes were drawn out strongly in Maclean's most influential work, his *Results*. Under the patronage of the Duke of Kent, Lord Grenville, and the Levant Company, Maclean had traveled to Turkey in late 1815 to study the plague. *Results*, the two-volume account of his research there, is usually seen as the catalyst for the first of the parliamentary inquiries. Lord Grenville presented *Results* to the Levant Company court, whose members, in turn, asked Grenville to present it on their behalf to the Prince Regent and request an inquiry.^54^

In the first volume, Maclean had introduced his theory of disease, the origin of the false doctrine of contagion, and the pernicious effects of belief in that doctrine. His second volume featured a vivid account of the several weeks he spent in a Pest Hospital, where he suffered an attack of the plague, and his remaining time in quarantine in Constantinople. He then set out again his theory of the disease and went into some detail on appropriate treatments. These were not changed in any significant respects from his earlier work. However, in contrast with his earlier work, *Results* focused strongly on quarantine and on the involvement of the Catholic Church in promoting the false doctrine of contagion.

The immediate utility of the antiquarantinist argument to Maclean lay in its appeal to his new patrons, merchants. Maclean was very eager to have *Results* published and, as I already mentioned, he had cultivated the patronage of Lord Grenville and the Levant Company. Designing his letters to them to appeal to the commercial concerns of a trading company, he advocated an inquiry into the practice of quarantine on the basis of his findings against contagion. *Results* itself devoted long passages not only to quarantine but to calculations of the cost of epidemics—a language of political economy upon which Maclean had begun to rely in his 1810 critique of the Army Medical Department.^55^

The essence of Maclean's attack on Catholicism, his idea of "papal conspiracy," was that the idea of contagion was fabricated by Pope Paul III and promulgated by his doctor, Fracastorius, to ensure that key council members would be quarantined because of a sickness and excluded from an important vote during the Council of Trent.^56^ Far from being idiosyncratic[Other P-555] to Maclean, it was first advanced by "The Explainer" in 1722^57^ and by Sir Richard Manningham in 1758.^58^ Participants in eighteenth-century contagion debates were aware of the theory.^59^ It is not clear when Maclean became aware of it, but by the time he wrote of his experiences in the Levant (in 1817) he had absorbed Manningham's argument and was further asserting that there was a "despotic" Catholic tendency whereby Catholic nations were obliged to adopt the doctrine of contagion as an "article of faith."^60^ He also asserted the existence of a medico-ecclesiastic alliance to explain the receipt of the doctrine in Protestant England under Henry VIII.^61^

The "conspiracy" formed a cornerstone of the "new and improved" anticontagionist theory Maclean began to promote in *Results*. Throughout *Results*, he railed against the "pious fraud"^62^ of contagion and the role of the Catholic Church in its propagation. In the pursuit of his argument, Maclean employed the "Mahommedan" religion as a contrast to the errors of Catholic nations regarding contagion. Further, he stated that the risk of plague was increased by predisposing factors that he discussed in terms of religion; in his opinion, simply being Catholic was a risk factor.^63^

Maclean's radicalism, commitment to antiquarantinism, and hostility to the Catholic Church are not in question. It is also possible that his commitment to "truth" accounts for his inclusion of his admission that he caught the plague while in Turkey. However, Maclean must have been aware of the likelihood that these sensational inclusions would arouse public interest. His decision to give prominence to those issues was almost certainly calculated. They won him the essential support of his powerful patrons and politicians like John Cam Hobhouse, allowed him to earn significant space in medical journals and the popular press, and gave him a profile he had not been able to achieve with his previous medical writings.[Other P-556]

## Maclean's Increasing Influence

Maclean's efforts resulted in the 1819 and 1824 parliamentary debates on quarantine. Those debates, and the reaction of the medical establishment, will be considered at the end of this section. It is important first to consider how a professionally unsuccessful physician like Maclean achieved such influence. In the previous section, I showed how Maclean developed his theories to appeal more effectively to politicians, merchants, and the public, garnering him a public profile. In this section, I will consider reactions to his theories and establish the contemporary perception of Maclean's importance. I will argue that Maclean's prominent anticontagionist profile continued to develop because of his ability to set the terms of debate and that the negative reaction of the medical establishment to Maclean was, in large part, a response to his location of the debate outside the medical sphere.

Even during the height of his notoriety, Maclean was not the only prominent anticontagionist in Britain. John Armstrong^64^ and Thomas Southwood Smith^65^ were other like-minded practitioners who received the attention of both the press and politicians, but they never became associated with the anticontagionist position in the same way as Maclean. Neither was he the first prominent anticontagionist in Britain; indeed the sensational aspects of his theory bore a striking similarity to those in publications of the 1720s.^66^

These factors might have been used by his opponents to diminish Maclean, but when his importance was questioned, it was not generally on these grounds.^67^ The most damning criticism leveled at him was one of irrelevancy. The *Medico-Chirurgical Review* put this position most baldly, stating that, "[Maclean's] lucubrations have long ceased to make the slightest impression on the medical profession"^68^; "volume after volume rolls from his prolific pen, and lies, in its turn, neglected by the public."^69^*The Lancet* took up a similar refrain, "now that Dr Maclean has ceased to bore the House of Commons."^70^

However, assertions of Maclean's irrelevance are contradicted by the pages of those very journals and by the popular press. Constant reference to him by both supporters and opponents of anticontagionism and the passion exhibited by his opponents belie such contemporary appraisals.

He was referred to by such names as "the Goliah [*sic*] of the non-contagionists," ^71^ "the Apostle of non-contagion,"^72^ and "this Magnus Apollo of the non-contagionists."^73^ His position was even sometimes referred to (not necessarily with approbation) as "Macleanism,"^74^ and his followers as "Macleananites."^75^ His prominence in the contagion debate could possibly be explained by his persistence or by the sheer volume of his written work. However, although these two factors are clearly relevant in accounting for his high profile, his prominence is also indicative of the influence he exerted on the direction of medical debate and the impact this had on important changes taking place in medical philosophy at the time.

Maclean's ability to create controversy and to push himself to the forefront of debate was a skill he had cultivated from the early days of his career. He had a passion for journalism and was experienced in using the press to advance his opinions. He claimed to have been the owner and publisher of a newspaper in India—a claim supported by Wellesley's observations about "the tribe of editors of newspapers" in Bengal, including "a most audacious and turbulent demagogue, named McLean."^76^ His flair for controversy and his medical evangelism came together upon his return to England, where, as we have seen, he took up the editorship of the *Medical Observer*.

The debates over quarantine also brought the issue of contagion to the public through the nonmedical press. *The Times*, the *Quarterly Review*, and the *Westminster Review* all gave extensive coverage to the topic over the period of the two parliamentary inquiries, 1819-26. A dominant feature of the nonmedical press was its cautious support for relaxation of quarantine, but opinions about Maclean varied widely. At one extreme[Other P-558] the *Quarterly Review* progressed from mildly disapproving in 1822, stating that "Dr Maclean has not dealt fairly with the subject,"^77^ to scathing in 1826, "we know not which to wonder at most, the mind of the man who uttered [Maclean's evidence], or the patience of the committee who could listen to [it]."^78^ The *Westminster Review* ran two articles in 1825 written by Southwood Smith that strongly endorsed Maclean's medical views and personal integrity.^79^*The Times* was a more moderate arbiter of the debate; it published John Mitchell's anticontagionist evidence from the 1819 inquiry,^80^ and in 1822, ran several extremely positive articles about Maclean's anticontagionist efforts in Spain.^81^ By 1825, *The Times* was arguing for a revision of the quarantine laws, stating that the "old doctrine of contagion has been a great deal shaken by modern inquiry and experience." ^82^ In that article, an anticontagionist position was promoted, but the paper also strongly advocated caution and "thorough investigation"^83^ of the matter. Common to all of this coverage was an aspiration to the relaxation of quarantine, with the degree of caution necessary to achieve that end as the dividing factor. Common also to these works is the pervasive presence of Maclean. He is at least mentioned in nearly every article, and even the *Quarterly Review* commented on his tenacious pursuit of publicity, stating that he had kept his "view of the subject incessantly before the public."^84^ It was his success in courting politicians and the public through the press that most angered the medical establishment.

Maclean was almost uniformly ridiculed by the medical press.^85^ However, the lack of favor he found there should be viewed in the context of the copious articles, letters, and editorials given over to discussion of his[Other P-559] theories. Demonstrating his ability to set the agenda, these articles usually dismissed Maclean but then set about engaging him on his own terms. The characteristic aspects of his theory—"papal conspiracy," the inability of contagious disease to strike twice, and the lack of fear of the plague in the Levant—were debated and meticulously picked apart by his detractors. This engagement indicates that the contagionists took Maclean more seriously than they allowed. The vitriol exhibited in these reviews also demonstrates the anger Maclean inspired in the medical establishment. This was the anger of a frustrated body incredulous at the very serious professional attention given to Maclean overseas and dismayed by the challenge to its authority he represented.

Maclean had always been taken more seriously abroad than in Britain. As I have already indicated, many practitioners in the service of the East India Company and others in the tropics held theories similar to Maclean's.^86^ His theories also found much favor in Spain during the 1820s, when he persuaded the Spanish Cortes to overturn its sanitary laws.^87^ Foreign journals also gave Maclean much consideration. His *View of the Science of Life* was reviewed, extensively if negatively, in the *New York Medical Repository* in 1798.^88^

Maclean spent a significant period of his professional life in Germany. In that country and in Italy, his works were taken very seriously. In 1825 his "papal conspiracy" theory (and its necessary corollary: the failure of the ancients to mention the existence of contagion) was considered and rebutted by two prominent physicians, the German C. F. H. Marx and Annibale Omodei of Italy.^89^ This overseas attention was particularly galling in Britain, where it was complained that, "while scarcely any one thinks Maclean's arguments worthy of a serious refutation, we find Americans, French, Germans, and Italians, setting themselves to investigate in the most elaborate manner, the various grounds for his opinions."^90^

Although this overseas attention was vexing to the British medical establishment, it was eclipsed by the threat to its authority that Maclean's chosen forum for debate represented. Maclean's decision to turn to the public and to the legislature implicitly removed the question of contagion from the medical sphere, thus eroding medicine's professional privilege and threatening the authority of the Royal College of Physicians.^91^

Nowhere was Maclean's influence or his attack on medical authority more pronounced than in the two parliamentary inquiries. The 1819 inquiry looked specifically into the question of the contagiousness of plague; the second in 1824 addressed the issue of quarantine but was required to consider contagion. The inquiries were accompanied by debates in the House of Commons. The medical establishment was well represented at these hearings. However, despite the "expert" status accorded medical practitioners in the inquiries, the ultimate decision on the question of contagion was to be made not by them but by politicians. Accordingly, a significant encroachment on the professional authority of medicine was in progress.^92^ Despite the weight given to expert witnesses, the questions posed by the inquirers were those of nonmedical men. In fact, as in the debate conducted in the medical journals, the questions investigated by the committees reflected the agenda set by Maclean in his *Results*.^93^

The 1819 Select Committee concluded that there was no evidence to support a change to the "received doctrine of contagion" but specifically stated that the question of quarantine was outside its remit.^94^ These findings do not accurately represent the proceedings of the committee, which investigated thoroughly the opinion of nearly every witness on the quarantine laws and found that the great majority supported their amendment.[Other P-561] Sir John Jackson chaired the inquiry and was clearly persuaded by the argument of the anticontagionists.^95^ He refused to sign the contagionist report of the committee and spoke against it in Parliament.^96^ Maclean appeared as both the first witness and the last. He was the only witness to be called twice, and his *Results* informed much of the questioning. He claimed in his critique of the inquiry to have actually drafted questions for Jackson but was disappointed that Jackson did not use them verbatim.^97^

Far from providing a subtext to the proceedings, the exclusive ability of medical practitioners to determine the question of contagion was raised by Jackson, and Maclean's and Mitchell's opinions were specifically sought on the issue. Maclean was given the floor in his second address to the committee and definitively stated, "In conclusion, I may observe, that the question of contagion in epidemic diseases, as acknowledged even by its advocates, is entirely one of fact, not of physic, of which all persons of a liberal education are as competent to judge as physicians."^98^

The 1824 Select Committee considered the effect of quarantine on the foreign trade of Britain but not the question of contagion, which was considered settled by the 1819 Report.^99^ Medical witnesses were consulted by this committee, but only contagionists, as the opinion of anticontagionists on quarantine regulations was considered a foregone conclusion. The Committee concluded that the quarantine system was too onerous and recommended that the length of quarantine be reduced and penalties for contravention made less harsh. Debates on the successful Quarantine Laws bill in Parliament in the following year were accompanied by a petition from Maclean introduced into Parliament by John Smith.^100^ Smith gave strong support to Maclean and advocated the view that "the question, as to its contagious or non-contagious quality, was not so much a question of science as a question of fact, on which any man who was in the habit of weighing testimony, was qualified to decide."^101^

The eminent naval physician Sir Gilbert Blane was a witness to both committees. He appears to have been deeply concerned by Maclean's attack on the profession, stating that the errors of the anticontagionists[Other P-562] exposed the "dignity of the profession" to "the sneers of the extra-professional part of the community" because those errors lay "open to the detection of the most ordinary and uncultivated minds."^102^ He feared that division within the profession would cause public health authorities to "ask the assistance of some members of the bench or the bar accustomed to weigh evidence, and investigate facts, or even of such plain men as compose juries, than medical men, having so much reason to suspect that our minds are warped by prejudice."^103^

The medical establishment responded to this attack on their authority through the press. Reflecting Maclean's central role, most commentary directly addressed him and was usually unflattering. Repeated complaints were made regarding Maclean's decision to take the debate to the public, "to whom it should be observed he always addresses himself,"^104^ and were usually accompanied by an observation that the public and the legislature were not qualified to decide "questions of which they must be necessarily ignorant."^105^ The structure of the inquiries was also criticized: "that our abstract is not more clear, has arisen from the very desultory manner in which the inquiry was conducted. The number of medical men examined was nineteen; only two of whom . . . deny the contagious nature of the plague. The non-contagionists are . . . to the contagionists as ten to one [*sic*]. But we feel confident that . . . the disproportion generally is much greater."^106^

Professional privilege was reasserted through statements explicitly alleging the inadequate standard of proof required by non-medical men to decide the question: "[Maclean's arguments are] the kinds of arguments that might do very well for a wrong-headed reformer in the House of Commons, but which must greatly injure a medical writer in the eyes of his brethren."^107^

Maclean's most influential supporter, Southwood Smith, was also criticized for suggesting that the debate should be determined outside the exclusive sphere of medical expertise, "while he denies medical men the right, or rather the capability of judging in their own concerns . . . he roundly asserts that contagion is not a question exclusively medical, but one of science, to be decided by facts. We would be glad to know[Other P-563] what question, among medical men, is not decided by facts?,"^108^ and for addressing his arguments to non-physicians who were "less likely to detect errors" in his reasoning.^109^

The medical press cast the worst possible light on Maclean's association with commercial interests, alleging that he was merely a puppet for "his retainers, the English merchants,"^110^ thus attempting to undermine his medical credibility: "the Macleananites' . . . science consists exclusively in . . . the balance of profit and loss."^111^ Perhaps sensing the almost universal support for an easing of the quarantine restrictions, the medical press made clear attempts to separate the questions of contagion and quarantine, arguing that Maclean had "retarded . . . desirable change" to the quarantine laws.^112^ Thus, the medical press also reasserted the exclusive authority of practitioners to decide the question: "We may be allowed to say, that we think the medical question of contagion has been unnecessarily mixed up with matters tending very much to bias a portion of the public against the doctrine . . . It has latterly taken too much of a commercial turn."^113^

The direction in which Maclean drove the debate—away from the exclusive authority of the medical profession—was characteristic of his anti-authoritarianism and belief in the availability of scientific knowledge to all^114^ and aroused the anger of the medical elite. However, this was not Maclean's only attack on that body.

## Contagion Debates and New Approaches to Medicine

A challenge to the exclusivity of medical expertise directly affected the Royal College of Physicians, whose prominent members led the opposition to Maclean. However, the contagion debates also encapsulated a more dangerous threat to that establishment, resulting from changes taking place in British medicine during this period as it underwent a shift[Other P-564] from a physiological to an ontological concept of disease.^115^ The confusion caused by the emergence of a new empirical approach to medicine and the struggle between old and new knowledge forms an important, and to date unexamined, aspect of the contagion debates of the 1820s. Christopher Lawrence states that during and following the Napoleonic Wars there was "intense conflict over the constitution of such things as anatomical, physiological and pathological facts and, more broadly, the method appropriate to the production of those facts and the theories the facts were used to sustain."^116^ In this section, I will demonstrate the contribution of the 1820s contagion debates to that conflict.

Much of the impetus behind new forms of knowledge during this period came from practitioners outside the college sphere. Many of these were overseas practitioners like Maclean, graduates of Scottish universities who had trained in military medical departments. W. F. Bynum has argued that these practitioners had an instrumental role in promoting the discussion and adoption of new ideas, particularly in relation to fever.^117^ The growing importance of these practitioners in Britain can be seen in the influence exerted by Anglo-Indian practitioners during debates over the contagiousness of cholera in the 1830s.^118^ Michael Durey has considered the use of evidence and knowledge systems in the cholera debates of the 1830s. Durey's analysis of those debates led him to conclude that, at that time, medical science was in a Kuhnian pre-paradigm state and that the consequent lack of a useful evidentiary system produced debates between the contagionist Royal College of Physicians and anticontagionists that were "inchoate, petty and increasingly shrill."^119^

A similar problem was manifest in the earlier debates of the 1820s, demonstrating that the effects of "paradigm conflict" were felt prior to the emergence of cholera as a real threat to Britain, and in the absence of consequent panic. Contrary to Durey's findings about the later debates,[Other P-565] the participants of the 1820s appear to have been conscious of their position, referring to "the unsettled nature of the laws of evidence in regard to medical inquiries"^120^ and the need to establish meaningful ground on which to engage in a conflict of ideas.

The relevance of overseas medical innovation to the debates of the early 1800s was grasped by Maclean, who for many years had been making attacks on the "monopoly of the College." Maclean argued that the College's antiquated membership policies excluded Scottish practitioners in particular and that its myopic view of medicine was obstructing the adoption of practices developed by the experimental approach fostered in the Scottish universities, the military, and overseas.^121^ At the time of the contagion debates, the work and practices of such practitioners had filtered back to Britain and begun to disturb medical orthodoxy. In addition, Maclean's influence on the direction of contagion debates and his passionate support for the empirical/experimental approach ensured that this issue was embedded in the considerations of the antagonists. The statements of witnesses to the inquiries and the writings of eminent physicians demonstrate a struggle on both sides of the debate to establish what type of evidence would be probative in not only this, but all medical debates, and what type of witness would be considered competent to give it.

Durey suggests that the practitioners of the College (who were nearly all contagionists) were united by the "antediluvian" education they received at Oxford and Cambridge, where "undue emphasis was still placed on the value of a literary education and a respect for ancient authority."^122^ In this context, we can see Maclean's assertion that the ancients knew nothing of "contagion" served two purposes; it supported his "papal conspiracy" theory but also fell firmly within traditional forms of medical argument to support his anticontagionist position. Reactions to his argument reveal the transitional state of medical knowledge. Usually practitioners articulated an intuition that debates about the ancients were meaningless, "to the opinions of the ancients on contagion, we do not think them of much value,"^123^ and asserted the value of empiric evidence: "we never, indeed, could see the very great importance of referring back to the ancients for evidence of that which passes before our own eyes."^124^ Yet those same practitioners still apparently felt compelled to engage with the proposition[Other P-566] and extensively set out references to contagion in classical works.^125^ Predictably, the College maintained most strongly the value of ancient texts, asserting in their report to Parliament on Maclean's *Results* that considerable evidence would be required to counterbalance the "weight of ages."^126^

These exchanges demonstrate the side-by-side existence of two probative systems and a medical profession that grasped the significance of the empirical model but was unable to let go of the old. This conflict was evident to the layperson, and in the 1819 inquiry, Jackson directly asked William Gladstone, surgeon to the Naval Asylum at Greenwich, whether he would "rather be governed by modern facts" or "historical reports?"^127^

As the medical establishment came to feel more threatened, their focus turned to the momentum of new ideas, and a need for caution was emphasized. An article in the *Edinburgh Medical and Surgical Journal* argued that the rash of "new" ideas and the wholesale rejection of time-honored precedent within medicine were causing severe consternation: "Does any one pant after eminence and distinction—let him attack opinions which have been long received, and which are sheltered under the authority of illustrious and venerable names."^128^ The question asked was: was novelty being pursued simply for its own sake? Maclean was particularly targeted by such commentators, who suggested quite plainly that his intention was to become personally famous through this device of "novelty" rather than to actually better medical practice.^129^

The majority of commentators did not reject these new ideas out of hand. Contagion and quarantine were perceived to be very important to the national interest, and the most frequent complaint in the press was dissatisfaction with the quality of argument from all parties. The contagionists were criticized for dealing in "fabulous" accounts of contagion lying wait for years in infected cobwebs and leather coats,^130^ and the anti[Other P-567] contagionists were accused of merely playing word games.^131^ The consensus was that nearly all medical practitioners, and particularly Maclean, approached the question full of prejudice.^132^ The parliamentary inquiries were not considered to have decided the question, and calls were made for *impartial* investigation.^133^ In Parliament, John Smith argued for the appointment of a commission "consisting of medical practitioners partly, and partly of men of general science and experience, charged to collect and examine into, and observe facts connected with the propagation of the plague."^134^

In efforts to achieve a resolution, practitioners began to express the debate as a clash between empiric and theoretical modes of reasoning, giving the medical establishment and many members of the college cause to be particularly alarmed.^135^ Even eminent "establishment" contagionists promoted this position. In his evidence for the 1824 inquiry, Augustus Bozzi Granville said clearly that he wanted his evidence to be confined to "practical, not theoretical" matters. He believed that the "theoretical" evidence given to the 1819 inquiry had done more harm than good and implied that the evidence of witnesses who "had never seen the plague. . . . and spoke merely from theoretical views" could lead to "no useful conclusion."^136^ The threat empiricism presented to the College lay in its valuing more highly the observations of practitioners who had personally observed and treated a disease. Given the importance of cholera and fevers during this period, such a value system would automatically count more highly the opinions of practitioners who had served overseas, where those diseases were more prevalent. According to Hume in parliamentary debate, he "would certainly prefer the opinions of those who had visited the countries in which the plague occasionally showed itself."^137^ Even Gilbert Blane, when considering the question, concluded that the two modes of reasoning should be given equal weight.^138^

Maclean's challenge to the British medical establishment was twofold. His attack on the privilege of the College of Physicians and the exclusivity of medical knowledge was overt, but the debate he engendered also tapped into more profound changes taking place in medical philosophy during this time. The debate on contagionism in the 1820s provided a forum in which the anxieties created by those changes could be articulated, throwing into sharp relief the evidentiary struggle between the two medical paradigms. The growing authority of medical practitioners, often Scottish and schooled outside the Oxbridge tradition, was also highlighted, giving the medical establishment further cause for concern and, no doubt, fuelling their animosity toward Maclean.

The questions Maclean raised about the availability of medical knowledge were closely related to the constitution of that knowledge. Maclean's construction of the debate allowed the clash of "theoretical" and "empirical" medical paradigms to be felt and explored. This conflict exposed an emerging preference for the evidence of those experienced in facing epidemic disease, experience that was usually gained overseas.

These two related challenges to medical authority had a profound effect on the reaction of the medical establishment to anticontagionism and to the quarantine debates. Quarantine and Catholicism provided Maclean with the means to take the debate away from the medical sphere. Quarantine also served as a tangible battlefield on which the two sides could engage, but the real issue in the minds of the medical antagonists was Maclean's challenge to the "old order" of the Royal College of Physicians and the future of the medical profession.[Other P-569]

## Footnotes

1
					Plague, a Contagious DiseaseQuarterly Review1825-182633218-257 on p. 256
				

2 Autobiographical details in Maclean's published works provide the following brief biography: Maclean was born around 1765 and by 1788 had obtained the degrees of physician and surgeon. He commenced practice as the surgeon of the East Indiaman, *William Pitt*. In the service of the company he traveled to Bengal, Jamaica, and the East Indies. He left the company's service in India and in 1796 worked in the Mariner's Ward of the Calcutta General Hospital. At this time he was also proprietor of a newspaper. In July 1798, he was ordered by Wellesley to leave India because of his criticism of a magistrate. After a year-long cruise he arrived in England and almost at once departed for Germany, where he remained throughout most of 1800 and 1801 seeking opportunities for the study of epidemic diseases. In 1801 he moved to Paris and was there during Napoleon's detention of the English in France in 1802. On proving he had not been in England for ten years, he was permitted to leave France. In 1804 he applied for a position with the Hospital Staff of the British Army and served in York Hospital Chelsea, Chelmsford, Gosport, and Cork until 1805. He left the service but was listed in *The Gazette* as "superseded for being absent without leave" and in the *Hue and Cry* as a "Deserter." In 1810 he began lecturing on the "Diseases of Hot Climates" under the sanction of the East India Company and in 1815 traveled to the Levant to "investigate the plague." From his return that year he agitated strenuously for reform of the quarantine laws, participating in both the 1819 and the 1824 parliamentary inquiries. In 1821 he was commissioned by the Spanish government to investigate the fever in Barcelona and was successful in persuading the Cortes to overturn Spain's sanitary laws. Publications and letters to periodicals from Maclean cease after 1826; see also HarrisonMarkMaclean, Charles (fl. 1788-1824) in Oxford Dictionary of National BiographyOxfordOxford University Press2004http://www.oxforddnb.com/view/article/17649 (accessed 2452006

3
					MacleanCharlesResults of an Investigation Regarding Epidemic and Pestilential Diseases vol. 1LondonThomas and George Underwood1817 MacleanCharlesResults of an Investigation Regarding Epidemic and Pestilential Diseases vol. 2LondonThomas and George Underwood1818
				

4 *Report from the Select Committee Appointed to consider the validity of the Doctrine of Contagion in the Plague*, Parliamentary Papers (hereafter referred to as PP), 1819, II.537; *Second Report from the Select Committee Appointed to consider the means of improving and maintaining The Foreign Trade of the Country*, PP, 1824, VI.165.

5
					AckerknechtErwinAnticontagionism Between 1821 and 1867Bull. Hist. Med.194822562-59318106184
				

6
					PellingMargaretCholera, Fever and English Medicine 1825-1865OxfordOxford University Press1978
				

7
					CooterRogerAnticontagionism and History's Medical Record in The Problem of Medical Knowledge: Examining the Social Construction of Medicine ed. WrightPeterTreacherAndrewEdinburghEdinburgh University Press1982 p. 87
				

8 See also BaldwinPeterContagion and the State in Europe 1830-1930CambridgeCambridge University Press1999 for a contrasting perspective on the issue of contagion.

9 See also MullettCharles. F.Politics, Economics and Medicine: Charles Maclean and Anticontagion in EnglandOsiris195210224-251 For a similar treatment of debates in France and America during the same period, see: Amory WinslowCharles-EdwardThe Conquest of Epidemic Disease, A Chapter in the History of IdeasWisconsinUniversity of Wisconsin Press1980 pp.193-223HeamanElspeth A.The Rise and Fall of Anticontagionism in FranceCan. Bull. Med. Hist.1995123-2510.3138/cbmh.12.1.311609097

10
					AckerknechtAnticontagionism (n. 5 p. 591
				

11
					PellingCholera (n. 6 p. 28
				

12
					CooterAnticontagionism (n. 7 p. 96
				

13
					CooterAnticontagionism (n. 7 p. 95
				

14 See DureyMichaelThe Return of the Plague—British Society and the Cholera 1831-2DublinGill & Macmillan1979 pp. 106-107MaglenKristaThe First Line of Defence: British Quarantine and the Port Sanitary Authorities in the Nineteenth CenturySoc. Hist. Med.20021541710.1093/shm/15.3.41312659089

15
					MacleanCharlesThe Affairs of Asia Considered in Their Effects on the Liberties of Britain2nd ed. LondonPrinted for the Author1806 p. 105
				

16 See, for example, MacleanCharlesAn Analytical View of the Medical Department of the British ArmyLondonJ. J. Stockdale1810 p. 59

17 See MacleanCharlesA View of the Science of Life; On the Principles Established in the Elements of Medicine, of that Late Celebrated John Brown MDMacleanCharlesYatesWilliamDover, N.H.Samuel Bragg1801 p. 122-147

18
					MacleanCharlesOn the State of Vaccination . . . LondonPrinted for the Author1810 p. 30
				

19
					MacleanA View (n. 17 preface; MacleanCharlesPractical Illustrations of the Progress of Medical ImprovementLondonPrinted for the Author1818 p. xxxvii

20 See, generally, BynumW. F.PorterRoy eds., Brunonianism in Britain and EuropeMed. Hist.1988 (Suppl. 848-52

21
					MacleanA View (n. 17 p. 20
				

22
					HarrisonMarkClimates and Constitutions: Health, Race, Environment and British Imperialism in India 1600-1850New DelhiOxford University Press1999 pp. 64 -72 although it should be noted that Maclean's principles for the prescription of mercury were different than those of his East Indian colleagues.

23 Yates entered the medical department of the East India Service as an assistant surgeon in 1795 and became surgeon in the course of his seniority. He served in the general hospital at Calcutta, was detached to Ceylon, and was eventually appointed garrison surgeon at Point de Galle, where he served for seven years. See Wellcome LibraryRAMC 1431: Box 305 Fifth Report of the Commissioners of Military Enquiry p. 190

24
					MacleanA View (n. 17
				

25 Maclean opened the body of every patient who died under his charge in the Calcutta General Hospital: MacleanA View (n. 17 p. 146

26
					MacleanPractical Illustrations (n. 19 pp. xii-xiii
				

27
					MacleanA View (n. 17 p. 13 see also MacleanPractical Illustrations (n. 19 p. xii

28
					MacleanA View (n. 17 pp. 54151 see also MacleanPractical Illustrations (n. 19 p. xii

29
					Fever-Contagion-QuarantineMedico-Chirurgical Review182521-23PMC507636229917730
				

30
					MacleanA View (n. 17 p. 152
				

31
					MacleanA View (n. 17 p. 155
				

32
					MacleanA View (n. 17 pp. 155178
				

33
					MacleanA View (n. 17 pp. 159-160
				

34
					MacleanA View (n. 17 p. 164
				

35
					MacleanA View (n. 17 pp. 179-181
				

36
					MacleanA View (n. 17 pp. 152-183 This section of the book contains Maclean's dissertation on "Epidemic and Pestilential Diseases." In this entire dissertation he mentions quarantine only briefly on p. 181.

37 Quarantine is mentioned briefly in his political work, An Excursion in France and Other Parts of the Continent of Europe . . .LondonT. N. Longman and C. Rees1804 p. 222 It receives no attention in his other medical works written before 1816, and I have not discovered any mention of quarantine in Maclean's letters to medical journals prior to that date.

38
					MacleanExcursion (n. 37 p. 21
				

39
					Maclean*Excursion* (n. 37 pp. 21-24
				

40
					MacleanAnalytical View (n. 16 pp. 149-158
				

41
					MacleanAnalytical View (n. 16 pp. 149-158 in this regard, Maclean strongly associated the Royal College of Physicians with the Army Medical Board and attacked both bodies for restricting entry to practice and advancement. See: MacleanCharlesOn the Monopoly of the College of PhysiciansMed. Observ.hereafter referred to as MO18071300-324MacleanCharlesDr Maclean's Second Letter on the Monopo1y of the College of Physicians18081345-387MacleanCharlesDr Maclean's Third Letter on the Monopo1y of the College of Physicians1808252-70Aretaeus (pseud.), On the Injurious Consequences to the ArmyMO18082155-160Aretaeus (pseud.), Aretaeus' Second LetterMO18082221-230AretaeusAretaeus' Third Letter1808MO2349-358

42
					MacleanCharlesTo the British Inhabitants of IndiaCalcutta1798 p. 17
				

43
					MacleanCharlesA View of the Consequences of Laying Open the Trade to India to Private ShipsPamphleteer18131199-204 MacleanCharlesRemarks on the Evidence Delivered Before Both Houses of Parliament, on the East-India Company's AffairsLondonBlack, Perry and Co.1813 p. 16
				

44
					MacleanAnalytical View (n. 16 p. 198
				

45 See Maclean's account of his correspondence with these persons and companies detailed throughout MacleanCharlesSpecimens of Systematic MisruleLondonH. Hay1820 also British LibraryAdd. 59265Dropmore Papers pp. 92-188 ff

46
					MacleanOn the Monopoly of the College of Physicians (n. 41 pp. 339-340; 1808278
				

47 See MO (n. 41

48 Immediately following MO18083

49
					MacleanAnalytical View (n. 16 p. 51 Maclean's reference to "the College" is to The Royal College of Physicians, which exerted significant power over the careers of medical practitioners in the army during this period because of its strong relationship with the Army Medical Department.

50 The term "medical establishment" is used in this paper to refer to the large group of contagionist practitioners who set themselves in opposition to Maclean. This group, dominated by prominent members of the Royal College of Physicians, was in general composed of successful and "respectable" practitioners who supported the system of medicine taught at Oxford and Cambridge.

51
					WrightJ. F.British Government Borrowing in Wartime, 1750-1815Econ. Hist. Rev.199952355
				

52
					ParryJonathan P.The Rise and Fall of Liberal Government in Victorian BritainLondonYale University Press1993chap. 1 see also HiltonBoydCorn, Cash, Commerce: The Economic Policies of the Tory Governments 1815-1830OxfordOxford University Press1977

53
					ColleyLindaBritons, Forging the Nation 1707-1837Yale University Press1992
				

54 Dropmore Papers (n. 45), f. 175.

55 See, for example, MacleanAnalytical View (n. 16 p. 16: "[it] would produce an annual saving at least to the extent of a third, if not half of the lives lost . . . in his Majesty's armies, and consequently of immense sums of money to the state." Other military medical practitioners such as Gilbert Blane, Thomas Trotter, and Robert Robertson were also embracing this style of argument during this period.

56 For a detailed explanation, see MacleanResults vol. 1 (n. 3 pp. 185-201

57 The Explainer (pseud.), Distinct Notions of the Plague With the Rise and Fall of Pestilential ContagionLondonJ. Peel1722 pp. 121-123

58
					ManninghamRichardA Discourse Concerning the Plague and Pestilential FeversLondonJ. Robinson1758 p. 32
				

59 See RussellPatrickA Treatise of the Plague Containing an Historical Journal and Medical Account of the Plague at Aleppo, in the years 1760, 1761 and 1762LondonG. G. J. and J. Robinson1791 p. 329: "this shows how groundless is the assertion that the notion of contagion took its rise in Europe no earlier than the 16th century, and how absurdly it has been ascribed merely to the political intrigue at the time of the Council of Trent."

60
					MacleanResults vol. 1 (n. 3 p. 205
				

61
					MacleanResults vol. 1 (n. 3 pp. 212357
				

62
					MacleanCharlesSuggestions for the Prevention and Mitigation of Epidemic and Pestilential DiseasesLondonThomas and George Underwood1817 p. 17
				

63
					MacleanResults vol. 1 (n. 3 p. 268
				

64 See Lectures on the Principles and Practice of Physic by Dr ArmstrongLancet18257193-204

65 See SmithThomas SouthwoodContagion and Sanitary LawsWestminster Rev.18253134-167 pp. 152 see also SmithThomas SouthwoodPlague—Typhus Fever—QuarantineWestminster Rev.18253499-530

66 The Explainer, Distinct Notions (n. 57PyeGeorgeA Short Discourse Concerning Pestilential Contagion Wherein Dr Mead's Notions Are Considered and RefutedLondonDarby1721

67 The derivative nature of Maclean's work was criticized by Hudson Gurney during debate in the House of Commons. Hansard Parliamentary Debates2nd ser. vol. 121825col. 1321

68
					Fever-Contagion-Quarantine (n. 29 p. 18PMC507636229917730
				

69
					Fever-Contagion-Quarantine (n. 29 p. 21PMC507636229917730
				

70
					Jemmy CoplandLancet1825877
				

71
					Report on Contagion of the PlagueEdinburgh Med. & Surg. J.182016109-124
				

72
					Letter to the Editor from A. B. GranvilleThe London Medical Gazette1830-18317106
				

73
					Jemmy Copland (n. 70 p. 77
				

74
					Dr Grattan's Report of the Dublin Fever HospitalLancet18259410
				

75
					Contagion-Plague-QuarantineMedico-Chirurgical Review18253292PMC507636229917730
				

76
					Wellesley to Henry Dundas, 21 March 1799 in Two Views of British India: The Private Correspondence of Mr Dundas and Lord Wellesley: 1798-1801 ed. IngramEdwardBathAdams and Dart1969 p. 235
				

77
					Contagion and QuarantineQuarterly Review182227532
				

78
					Plague, a Contagious Disease (n. 1 p. 256
				

79
					SmithSouthwoodContagion and Sanitary Laws (n. 65 SmithSouthwoodPlague —Typhus Fever—Quarantine (n. 65
				

80
					Parliamentary Papers—Evidence Before a Select Committee of the House of CommonsThe Times Digital Archivehereafter referred to as Times28101819 p. 3 and Parliamentary Papers—Report from the Select Committee on the Doctrine of Contagion in the PlagueTimes611820 p. 2 both available at http://infotrac.galegroup.com (last accessed 2452006

81
					Correspondence from Spain, Bilboa, Jan 3Times1711822 p. 3 and Correspondence from Spain—Cadiz, Jan 1Times2211822 p. 2 both available at http://infotrac.galegroup.com (last accessed 2452006

82
					We are glad to see a bill in progress for the revision of the Quarantine LawsTimes241825 p. 2 available at http://infotrac.galegroup.com (last accessed 2452006
				

83
					The subjoined paragraph is from an evening paperTimes641825 p. 2 available at http://infotrac.galegroup.com (last accessed 2452006
				

84
					Plague, a Contagious Disease (n. 1 p. 240
				

85 For an anomalous, contrasting view, see Foreign DepartmentLancet18256277

86 See, for example, Wellcome Library, *RAMC 210.3* (Papers of William Fergusson), "Documents regarding a ship of Black Recruits . . . ," in which Fergusson cites Maclean's writings as evidence supporting Fergusson's claim that yellow fever was not contagious.

87
					Correspondence from Spain, Bilboa (n. 81 Correspondence from Spain—Cadiz (n. 81 MacleanCharlesEvils of Quarantine Laws, and Non-Existence of Pestilential Contagion . . .LondonThomas and George Underwood1824 pp. 110-320
				

88
					New York Medical Repository17981531-5401798269-71
				

89
					MarxC. F. H.On the Origin of the Doctrine of ContagionD. R. MarxCarlsruhe and Barden1824 and Contagio cognito agli storici e filosofi antichi, ignorato dai medici Greci, Latini, Arabi, non immaginato da Papa Paolo III—Errore di Maclean—Dottrina del contagio fondata nel secolo quinto decimo da Marsilio Ficino e da Alessandro BenedettoAnnali Universali di Medicina compilati dal Signor Dottore Annibale Omodei vol. xxiiMilan1822 both as reviewed in Critical AnalysisEdinburgh Med. & Surg. J.18252499-143PMC582875930331969

90
					Critical Analysis (n. 89 p. 102
				

91 Although the Royal College of Physicians did exert significant power during this period and had the ear of the crown and Parliament on medical questions, I do not suggest that it in fact had exclusive authority on medical questions but, rather, that it asserted that authority for itself and for practitioners it considered well educated.

92 Some parliamentary consideration of medical debates had begun during the Napoleonic Wars; see, for example, Fifth Report of the Commissioners (n. 23 See also Proceedings and Report of a Special Medical Board Appointed by His Royal Highness the Commander in Chief, and The Secretary at War to Examine the State of the Hospital at the Military Depot in the Isle of WightL. B. Seeley, London1808 and Report from the Select Committee on the Ophthalmic HospitalParliamentary Papers, Reports from Committees1821IV335

93 For an account of the workings of Parliament and select committees in this period, see InnesJoannaLegislation and Public Participation 1760-1830 in The British and Their Laws in the Eighteenth Century ed. LemmingsDavidWoodbridge, SuffolkBoydell Press2005 pp. 121-130

94
					Report from the Select Committee (n. 4 p. 540
				

95 Jackson was born the son of a surgeon in Jamaica in 1763. He entered Parliament in 1806 as Member for Dover. In April 1807 he became a director of the East India Company. For further details, see his biographical entry in ThorneR. G. ed., The History of Parliament: House of Commons 1790-1820LondonSecker and Warburg1986

96
					Hansard1st ser. vol. 401819col. 1133-34
				

97
					MacleanSpecimens of Systematic Misrule (n. 45 p. 166
				

98
					Report from the Select Committee (n. 4 p. 97
				

99
					Second Report from the Select Committee (n. 4
				

100
					Hansard2nd ser. vol. 121825col. 993-96
				

101
					Hansard2nd ser. vol. 121825 p. 1315
				

102
					BlaneGilbertElements of Medical LogickLondonT. & G. Underwood1819 p. 181
				

103
					BlaneGilbertElements of Medical LogickLondonT. & G. Underwood1819 p. 182
				

104
					Fever in IrelandLancet182610721
				

105
					Fever in IrelandLancet182610 p. 721
				

106
					Report on Contagion of the Plague (n. 71 p. 122
				

107
					Fever-Contagion-Quarantine (n. 29 p. 20PMC507636229917730
				

108
					Reviews from the WestLancet18256336
				

109
					Plague, a Contagious Disease (n. 1 p. 240
				

110
					Jemmy Copland (n. 70 p. 77 see also Reviews from the WestLancet18257115

111
					Contagion-Plague-Quarantine (n. 75 p. 292
				

112
					Fever-Contagion-Quarantine (n. 29 p. 21PMC507636229917730
				

113
					Reviews from the West (n. 110 p. 115
				

114 Pelling considers this aspect of Maclean's agenda to be characteristic of the "Radical belief in scientific knowledge as pre-eminently, if not definitively, available to all": Cholera (n. 6 p. 29

115 See HamlinChristopherPublic Health and Social Justice in the Age of Chadwick, Britain 1800-1854CambridgeCambridge University Press1998 pp. 53-58KondratasRamunasThe Brunonian Influence on the Medical Thought and Practice of Joseph Frank in BynumW. F.PorterRoy eds., Brunonianism in Britain and EuropeMed. Hist.1988Suppl. 875

116
					LawrenceChristopherMedicine and the Making of Modern Britain 1700-1920LondonRoutledge1994 p. 29
				

117
					BynumW. F.Cullen and the Study of Fevers in Britain, 1760-1820 in BynumW. F.NuttonVivian eds., Theories of Fever from Antiquity to the EnlightenmentMed. Hist.1981 Suppl. 1 see also HarrisonMarkNetworks of Knowledge: Rethinking Science and Medicine in Early Colonial Indiaunpublished paper2004

118
					BaldwinContagion (n. 8 p. 39 AckerknechtAnticontagionism (n. 5 p. 575
				

119
					DureyReturn of the Plague (n. 14 p. 110
				

120
					Critical Analysis (n. 89 p. 100
				

121 See MO (n. 41

122
					DureyReturn of the Plague (n. 14 p. 108
				

123
					Report on Contagion of the Plague (n. 71 p. 122
				

124
					History of the Doctrine of ContagionMedico-Chirurgical Review18253578-583
				

125
					Report on Contagion of the Plague (n. 71 p. 122 see also Critical Analysis (n. 89 pp. 99-143Contagion and Quarantine (n. 77 pp. 351-352Reviews from the West (n. 108 p. 340 Letter from Sir Gilbert Blane to John Cam Hobhouse, 4 April 1825, British Library, Add. 36461, f. 59-60; MacmichaelW.A Brief Sketch of the Progress of Opinion upon the subject of Contagion; With Some Remarks on QuarantineLondonJohn Murray1825 p. 16

126
					Report from the Select Committee (n. 4 p. 100
				

127
					Report from the Select Committee (n. 4 p. 29
				

128
					Critical Analysis (n. 89 p. 100
				

129
					Critical Analysis (n. 89 pp. 100-103 History of the Doctrine of Contagion (n. 124 p. 578-583 HancockThomasResearches Into the Laws and Phenomena of Pestilence . . .LondonWilliam Phillips1821
				

130 Report from the Select Committee (n. 4), evidence of John Mitchell, M.D., p. 90; Reviews from the West (n. 110 p. 116

131
					Reviews from the West (n. 108 pp. 336-338 Reviews from the West (n. 110 p. 116 Fever-Contagion-Quarantine (n. 29 p. 21PMC507636229917730 The subjoined paragraph is from an evening paper (n. 83
				

132
					Plague, a Contagious Disease (n. 1 p. 239 Review of Tully and HancockMedico Chirurgical Review18212570
				

133
					We are glad to see (n. 82
				

134
					Hansard2nd ser. vol. 131825col. 603
				

135 The division between these two ways of knowing was, in practice, blurred. However, as the medical practitioners involved in these debates themselves employed those two categories as broad descriptors of their positions, I have adopted them in this article.

136
					Second Report from the Select Committee (n. 4 pp. 237-238
				

137
					Hansard2nd ser. vol. 121825col. 1326
				

138
					BlaneElements (n. 102 pp. 9296
				

